# Toward fully algorithmic care for type 1 diabetes: The case for AI autonomy, and a recalibrated role for the diabetologist in an ageing type 2 diabetes population

**DOI:** 10.1371/journal.pdig.0001734

**Published:** 2026-09-18

**Authors:** Mounzer Maaz, Samar Maaz, Islam Maaz, Randa Dardari, Helene Dardari, Dured Dardari

**Affiliations:** 1 Universitatea de Medicină și Farmacie „Carol Davila”, București, Romania; 2 Faculty of Medicine, University of Aleppo, Aleppo, Syria; 3 Pediatric Unit, Montrouge, France; 4 Diabetology Department, Centre Hospitalier Sud Francilien, Corbeil-Essonnes, France; 5 Laboratoire de Biologie de l’Exercice pour la Performance et la Santé, Université d’Évry, Institut de Recherche Biomédicale des Armées, Université Paris-Saclay, Évry, France; 6 Kremlin-Bicêtre Medical School, Université Paris-Saclay, Le Kremlin-Bicêtre, France; Lahore College for Women University, PAKISTAN

## Abstract

Artificial intelligence can already automate selected diabetes tasks, but the evidence is uneven. Automated insulin delivery is established in type 1 diabetes; in type 2 diabetes, validated screening and insulin-adjustment tools support a tiered, human-governed model rather than wholesale autonomy.

## Why episodic care cannot scale

**Fig 1:**
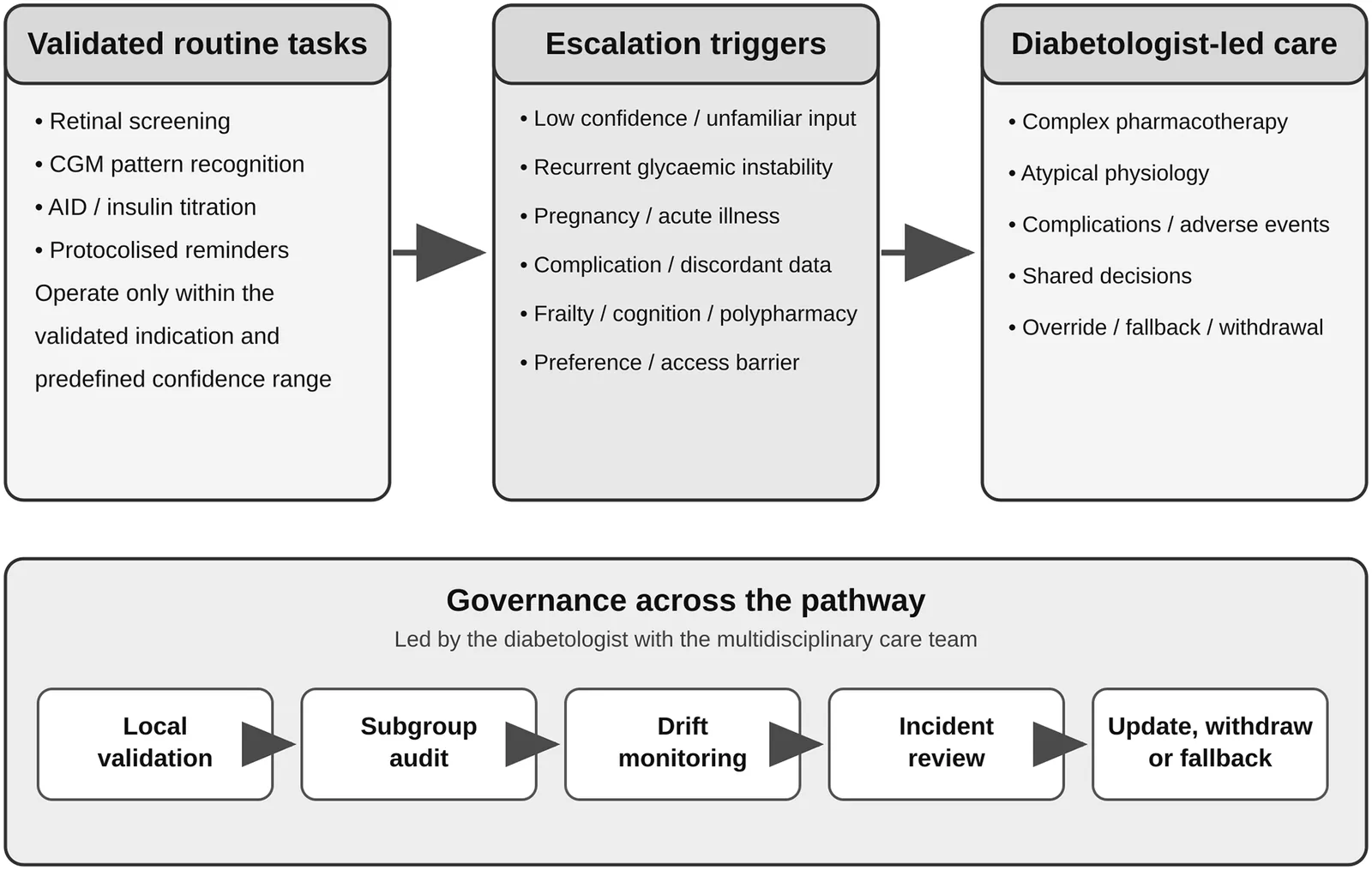
Conceptual task-allocation framework for algorithmic diabetes care. This schematic is not a time-series or population-based graph. Validated tools manage protocolised, high-volume tasks. Low confidence, clinical complexity, complications or patient preference trigger escalation to the diabetologist, who also leads local validation, subgroup audit, drift monitoring, incident review and equitable fallback pathways. AID, automated insulin delivery; CGM, continuous glucose monitoring.

In 2024, an estimated 589 million adults aged 20–79 years were living with diabetes, a total projected to reach 853 million by 2050 [1]. Guidelines call for regular reassessment and timely treatment modification [2], yet intensification in type 2 diabetes (T2D) can lag for years [3]. Artificial intelligence (AI) should therefore be compared not with an idealised, continuously available specialist, but with episodic care constrained by workforce, access and delay. Fig 1 presents a tiered allocation of validated routine tasks and specialist exceptions.

## Type 1 diabetes: Automation as standard

Type 1 diabetes (T1D) requires repeated insulin decisions that are well suited to closed-loop control. In the iDCL trial, automated insulin delivery (AID) increased time in range from 61% to 71% over six months without increasing severe hypoglycaemia [4]. In ADAPT, AID lowered HbA1c by 1.4%, compared with 0.2% for intensive multiple daily injections plus continuous glucose monitoring [5]. Benefit and safety also extend to young children [6]. Current standards therefore position AID as the preferred insulin-delivery approach for people with T1D who can use it safely [7].

This does not remove clinicians; it changes the default. For stable T1D, the diabetologist should concentrate on education, shared decision-making, onboarding, structured review and event-triggered care: pregnancy, intercurrent illness, recurrent hypoglycaemia, technology failure, developmental transition, psychosocial distress or complications. Routine manual adjustment of basal rates, insulin-to-carbohydrate ratios and correction factors should become less common when a certified AID system is functioning well.

## Type 2 diabetes: Task-specific evidence, not blanket autonomy

The burden of T2D is rising rapidly in adults aged 55 years and older [8], many of whom have multimorbidity, frailty, cognitive impairment or polypharmacy [9]. Here, the evidence for AI is narrower than in T1D and remains task-specific. Autonomous retinal AI has prospective evidence: IDx-DR achieved 87.2% sensitivity and 90.7% specificity for referable diabetic retinopathy in primary care [10], while DeepDR Plus predicted progression and supported risk-based screening intervals [11].

Evidence is also emerging for insulin adjustment. In 319 adults with insulin-treated T2D, AID reduced HbA1c by 0.9 percentage points, compared with 0.3 in participants continuing their pretrial insulin regimen with CGM, and increased time in range by an adjusted 14 percentage points [12]. In a 149-participant inpatient trial, an AI-assisted insulin titration system was non-inferior to senior endocrinologists over five days [13]. Current standards recommend offering AID to adults with T2D using multiple daily injections who can use it safely [7]. These findings justify automated or AI-assisted delivery for defined, validated tasks—not autonomous control of all T2D care. Non-insulin medication selection, cardiorenal prioritisation, bariatric pathways, frailty, cognition, polypharmacy and social context still require clinical judgement.

## Repositioning the diabetologist and governing risk

Algorithmic care introduces bias, calibration drift, out-of-distribution inputs, automation bias, cybersecurity and interoperability failures, liability gaps, and exclusion through cost or limited digital literacy [14]. The response is not uncritical adoption or reflex rejection, but explicit governance. Diabetologists should define eligibility, contraindications, confidence thresholds and escalation triggers; validate tools locally; audit outcomes by age, sex, ethnicity and socioeconomic position; monitor drift; investigate incidents; preserve human override and patient choice; and maintain safe non-digital fallback pathways. In low-resource settings, deployment must not make care contingent on smartphones, broadband, proprietary subscriptions or uninterrupted device supply.

At the bedside, specialist time should be concentrated where uncertainty or the consequence of error is greatest: atypical physiology, discordant data, recurrent adverse events, complications, complex pharmacotherapy and value-sensitive decisions. Human-in-the-loop allocation models formalise this principle by directing scarce human attention to cases with greater uncertainty or higher stakes [15]. Implementation should be staged through prospective validation, regulatory authorisation, guideline-concordant protocols, transparent audit logs, downtime plans and equitable reimbursement. Where these conditions are absent, conventional evidence-based care remains the standard.

## Conclusion

AI is not a universal substitute for the diabetologist. In T1D—and increasingly in appropriately selected insulin-treated T2D—validated AID can become the primary day-to-day therapeutic interface within guideline-concordant, clinician-governed care. In broader T2D, AI can be the first layer only for validated screening, triage and protocolised adjustment. The diabetologist’s value shifts towards onboarding, exceptions, complexity and stewardship. The goal is not fewer doctors, but better use of specialist judgement.

## References

[1] Genitsaridi I, Salpea P, Salim A, Sajjadi SF, Tomic D, James S, et al. 11th edition of the IDF Diabetes Atlas: global, regional, and national diabetes prevalence estimates for 2024 and projections for 2050. Lancet Diabetes Endocrinol. 2026;14(2):149–56. doi: 10.1016/S2213-8587(25)00299-2 41412135

[2] Davies MJ, Aroda VR, Collins BS, Gabbay RA, Green J, Maruthur NM, et al. Management of hyperglycaemia in type 2 diabetes, 2022. A consensus report by the American Diabetes Association (ADA) and the European Association for the Study of Diabetes (EASD). Diabetologia. 2022;65(12):1925–66. doi: 10.1007/s00125-022-05787-2 36151309 PMC9510507

[3] Khunti K, Wolden ML, Thorsted BL, Andersen M, Davies MJ. Clinical inertia in people with type 2 diabetes: a retrospective cohort study of more than 80,000 people. Diabetes Care. 2013;36(11):3411–7. doi: 10.2337/dc13-0331 23877982 PMC3816889

[4] Brown SA, Kovatchev BP, Raghinaru D, Lum JW, Buckingham BA, Kudva YC, et al. Six-month randomized, multicenter trial of closed-loop control in type 1 diabetes. N Engl J Med. 2019;381(18):1707–17. doi: 10.1056/NEJMoa1907863 31618560 PMC7076915

[5] Choudhary P, Kolassa R, Keuthage W, Kroeger J, Thivolet C, Evans M, et al. Advanced hybrid closed loop therapy versus conventional treatment in adults with type 1 diabetes (ADAPT): a randomised controlled study. Lancet Diabetes Endocrinol. 2022;10(10):720–31. doi: 10.1016/S2213-8587(22)00212-1 36058207

[6] Wadwa RP, Reed ZW, Buckingham BA, DeBoer MD, Ekhlaspour L, Forlenza GP, et al. Trial of hybrid closed-loop control in young children with type 1 diabetes. N Engl J Med. 2023;388(11):991–1001. doi: 10.1056/NEJMoa2210834 36920756 PMC10082994

[7] American Diabetes Association Professional Practice Committee for Diabetes. 7. Diabetes technology: Standards of Care in Diabetes—2026. Diabetes Care. 2026;49(Suppl 1):S150–65. doi: 10.2337/dc26-S007 PMC12690173 41358889

[8] Yu X, Kan C, Zhang K, Zhang X, Ren J, Chen J, et al. Global epidemiology and burden of type 2 diabetes in adults aged 55 and older: insights from 1990 to 2021. Ther Adv Endocrinol Metab. 2025;16:20420188251362011. doi: 10.1177/20420188251362011 40791828 PMC12336403

[9] Hashemi R, Rabizadeh S, Yadegar A, Mohammadi F, Rajab A, Karimpour Reyhan S, et al. High prevalence of comorbidities in older adult patients with type 2 diabetes: a cross-sectional survey. BMC Geriatr. 2024;24(1):873. doi: 10.1186/s12877-024-05483-3 39448921 PMC11515473

[10] Abràmoff MD, Lavin PT, Birch M, Shah N, Folk JC. Pivotal trial of an autonomous AI-based diagnostic system for detection of diabetic retinopathy in primary care offices. NPJ Digit Med. 2018;1:39. doi: 10.1038/s41746-018-0040-6 31304320 PMC6550188

[11] Dai L, Sheng B, Chen T, Wu Q, Liu R, Cai C, et al. A deep learning system for predicting time to progression of diabetic retinopathy. Nat Med. 2024;30(2):584–94. doi: 10.1038/s41591-023-02702-z 38177850 PMC10878973

[12] Kudva YC, Raghinaru D, Lum JW, Graham TE, Liljenquist D, Spanakis EK, et al. A randomized trial of automated insulin delivery in type 2 diabetes. N Engl J Med. 2025;392(18):1801–12. doi: 10.1056/NEJMoa2415948 40105270

[13] Ying Z, Fan Y, Chen C, Liu Y, Tang Q, Chen Z, et al. Real-time AI-assisted insulin titration system for glucose control in patients with type 2 diabetes: a randomized clinical trial. JAMA Netw Open. 2025;8(5):e258910. doi: 10.1001/jamanetworkopen.2025.8910 40332936 PMC12059970

[14] Sheng B, Pushpanathan K, Guan Z, Lim QH, Lim ZW, Yew SME, et al. Artificial intelligence for diabetes care: current and future prospects. Lancet Diabetes Endocrinol. 2024;12(8):569–95. doi: 10.1016/S2213-8587(24)00154-2 39054035

[15] Das S, Nikolaev A. Using Game-theoretic Value Of Information For Sequential Allocation Of Human Resources In Human-in-the-loop. In: 2024 IEEE Conference on Cognitive and Computational Aspects of Situation Management (CogSIMA), 2024. 107–13. 10.1109/cogsima61085.2024.10553768

